# Left ventricular mass estimation by real-time 3D echocardiography favourably competes with CMR in congenital left ventricular disease

**DOI:** 10.1038/s41598-019-48375-y

**Published:** 2019-08-15

**Authors:** Miriam Michel, Wael Shabanah, Hermann Körperich, Andrea Kelter-Klöpping, Andreas Entenmann, Anca Racolta, Kai Thorsten Laser

**Affiliations:** 1 Department of Pediatrics, Medical University of Innsbruck, Innsbruck, Austria; 2 Center for Congenital Heart Defects, Heart and Diabetes Center North-Rhine Westphalia, Ruhr-University of Bochum, Bad Oeynhausen, Germany; 3 Institute for Radiology, Nuclear Medicine and Molecular Imaging, Heart and Diabetes Center Northrhine-Westphalia, Ruhr-University of Bochum, Bad Oeynhausen, Germany

**Keywords:** Cardiology, Magnetic resonance imaging, Three-dimensional imaging, Ultrasonography, Congenital heart defects

## Abstract

Assessment of left ventricular mass (LVM) is important in the evaluation of patients with congenital heart disease (CHD) and cardiac magnetic resonance imaging (CMR) is the gold standard. Recent software allows LVM calculation by real-time 3-dimensional echocardiography (RT3DE). We investigated the impact of different software analysis tools on LVM determination by CMR or RT3DE in a cohort of patients with heterogeneous left ventricular (LV) disease. 37 subjects (17 patients, mean age 18.7 y; 20 controls, mean age 13.2 y) underwent CMR and RT3DE. CMR LVM and RT3DE calculations were done using two different LV-analysis software packages for each modality: CMR i) customized software “CMR HDZ”, CMR ii) “CMR ISP”; RT3DE i) “Toshiba”, RT3DE ii) “Tomtec”, 4D LV-Analysis Version 3.1 (built 3.1.0.258661). Intra- and interobserver variabilities were calculated. Only RT3DE-derived LVM showed significant software-dependent differences. RT3DE-derived LVM (both softwares) was significantly higher than CMR-derived LVM (both softwares). The two different methods and four evaluation software packages for LVM assessment were well correlated with each other. Intra- and interobserver variability of LVM as assessed by each single modality or software was low. Despite software dependency and overestimation of RT3DE-assessed LVM by 5 to 10%, RT3DE still competes with the gold standard, CMR, even in patients with various forms of LV disease. The use of optimized software, especially for RT3DE, should improve the accuracy of LVM assessment, overcoming LVM overestimation.

## Introduction

Non-invasive assessment of left ventricular mass (LVM) is important for monitoring patients with congenital heart disease (CHD). Up to now the gold standard for mass assessment has been cardiac magnetic resonance imaging (CMR)^1^. While standard-formula based mass estimation by 2-dimensional (2D) echocardiography risks inaccuracies, new software developments allow real-time 3-dimensional echocardiography (RT3DE)- based calculation of LVM^2,3^. Since particularly in patients with cardiac disease, *e.g*., CHD, clinical decision-making relies more and more on imaging data, the comparability of imaging results often is important not only intrainstitutionally but also interinstitutionally. Confounders like underlying cardiac malformation, treatment modality, type of hardware or software used, and the specific training background of the individual who performs the examination and analysis must be taken into account^1,4^. The influence of different software for LVM in pediatric, adolescent, and adult patients with heterogenous congenital LV disease, in particular, has not been addressed. Due to bed-side availability, low cost, short examination time, and lack of need for sedation with RT3DE, it is important to know how RT3DE competes with the gold standard, CMR.

Thus we investigated the impact of different analysis software systems on the reproducibility of CMR (as reference) and of RT3DE in measuring LVM in CHD patients with heterogeneous LV disease.

## Results

Due to poor image quality or incomplete volume capture in RT3DE studies, 2 patients (female, 11 years [y], double outlet right ventricle with pulmonary stenosis; female, 2 months, double aortic branch) and 1 control had to be excluded from evaluation, resulting in 17 patients (mean age ± standard deviation (SD) = 18.7 ± 16.2 y; range 0.1 to 72.9 y, 7 female) and 20 healthy individuals (13.2 ± 3.4 y; range 7.1 to 19.4 y, 9 female) included into the study. For detailed participant characteristics and diagnoses see Table 1.

**Table 1:** Patients’ diagnoses.

Patient diagnosis	n
Tetralogy of Fallot	4
Pulmonary atresia, intact interventricular septum (Fontan)	1
Transposition of great arteries (Mustard, morphologically LV)	1
Pulmonary stenosis, pulmonary regurgitation	3
Aortic stenosis, aortic regurgitation, aortic valve replacement	3
Aortic coarctation	1
Atrial septal defect, secundum type	2
Ventricular tachycardia	2

LV, left ventricle; SD, standard deviation; n, number of patients/controls.

**Figure 1:**
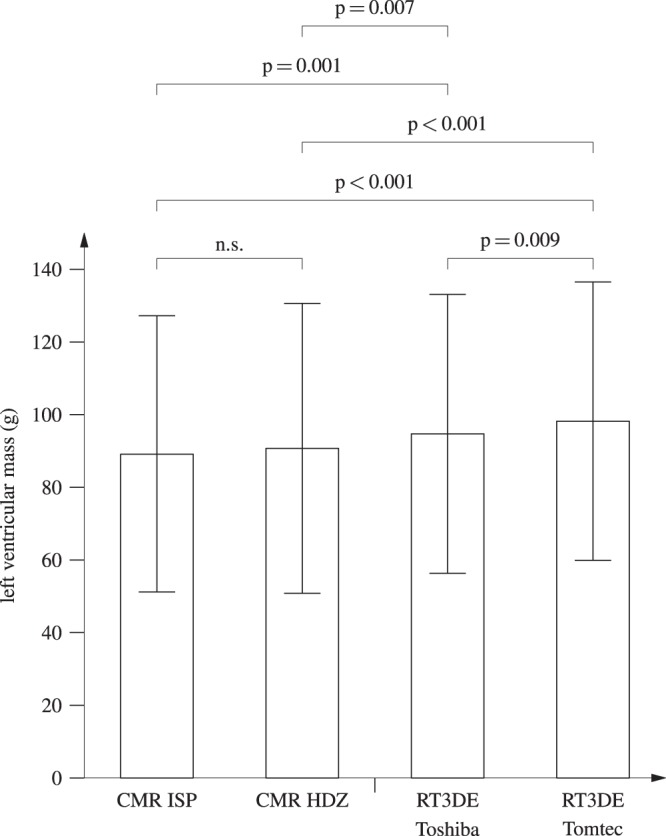
Left ventricular mass (LVM, absolute values) by type of assessment (CMR vs. RT3DE) and software used. CMR ISP: Cardiac magnetic resonance, IntelliSpace Portal; CMR HDZ: Cardiac magnetic resonance HDZ MR-Tools; RT3DE, Real-time 3-dimensional echocardiography. Boxes indicate mean LVM with error bars showing standard deviation. The p-values indicate statistically significant differences in mean LVM.

Mean CMR-derived LVM was 89.2 ± 38.0 g (mean ± SD) (“CMR ISP”) and 90.7 ± 39.9 g (“CMR HDZ”). LVM did not differ significantly between the software packages (Fig. 1; p = 0.235). Mean RT3DE-derived LVM was 98.2 ± 38.3 g (“Tomtec”) and 94.7 ± 38.4 g (“Toshiba”). RT3DE-derived LVM (“Tomtec”) was significantly higher than RT3DE-derived LVM (“Toshiba”) (+3.7%; p = 0.009). RT3DE-derived LVM was significantly higher than CMR-derived LVM (RT3DE LVM +5 to 10%, p < 0.01).

All intra-class coefficients (ICC) comparing the two different methods and evaluation software packages for LVM assessment were >0.97 (Table 2).

**Table 2:** Intraclass correlation coefficients (ICC) of left ventricular mass as assessed by cardiac magnetic resonance imaging or real-time 3-dimensional echocardiography, and by software tool.

	CMR HDZ	CMR ISP	RT3DE Toshiba
CMR ISP	0.982	—	—
RT3DE Toshiba	0.975	0.971	—
RT3DE Tomtec	0.979	0.979	0.980

CMR ISP: Cardiac magnetic resonance, IntelliSpace Portal; CMR HDZ: Cardiac magnetic resonance HDZ MR-Tools; RT3DE, Real-time 3-dimensional echocardiography.

**Figure 2:**
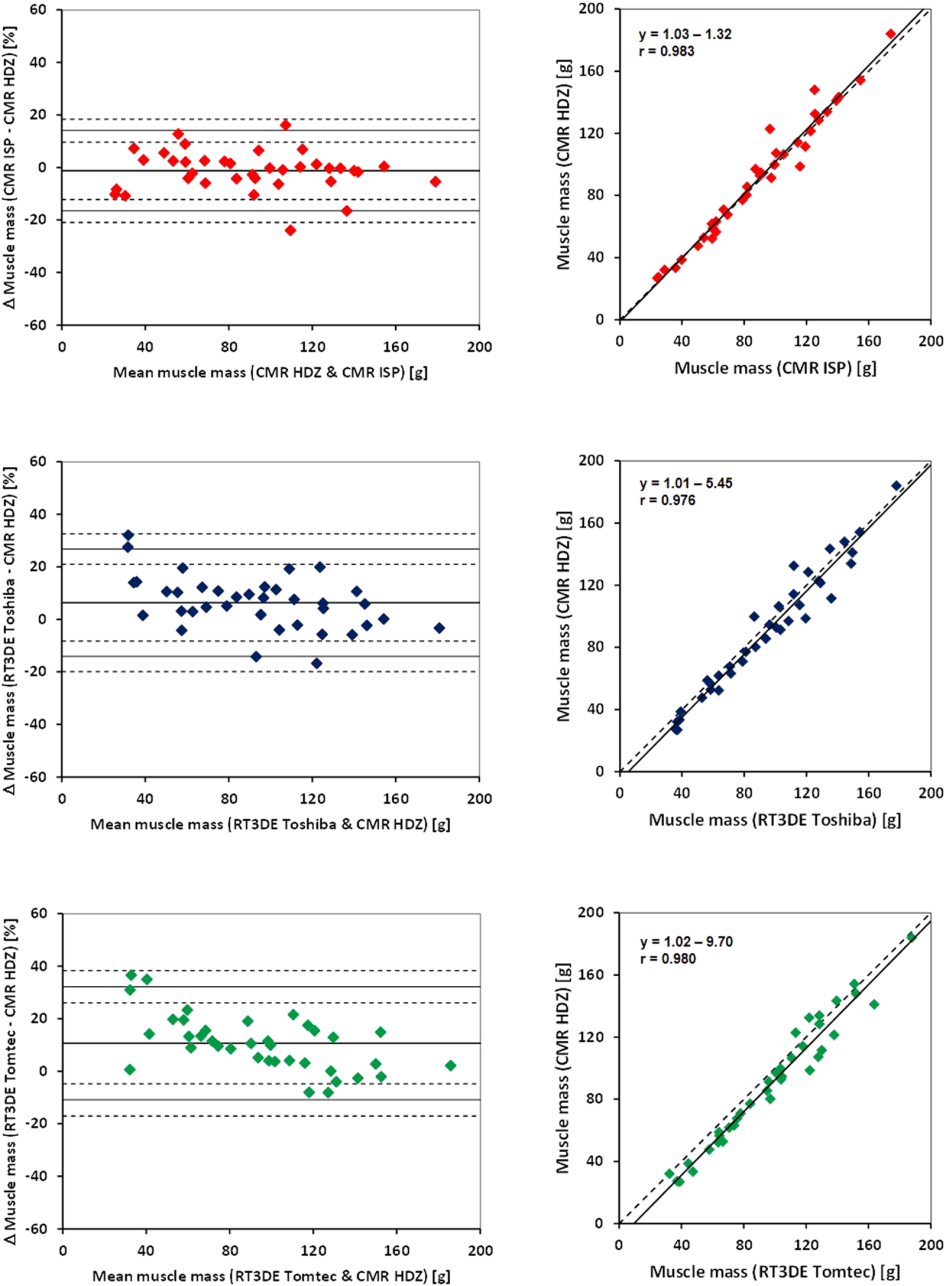
Right column: Bland and Altman plots for the comparison of left ventricular mass by type of assessment (cardiac magnetic resonance imaging *vs*. real-time 3-dimensional echocardiography) and software used (abscissa: mean left ventricular mass (absolute value), ordinate: differences between 2 measures [%]). Left column: Correlation between left ventricular masses (absolute values) determined by 2 types of assessment (cardiac magnetic resonance imaging *vs*. real-time 3-dimensional echocardiography) and 2 types of software. CMR ISP: Cardiac magnetic resonance, IntelliSpace Portal; CMR HDZ: Cardiac magnetic resonance HDZ MR-Tools; RT3DE, Real-time 3-dimensional echocardiography.

**Figure 3:**
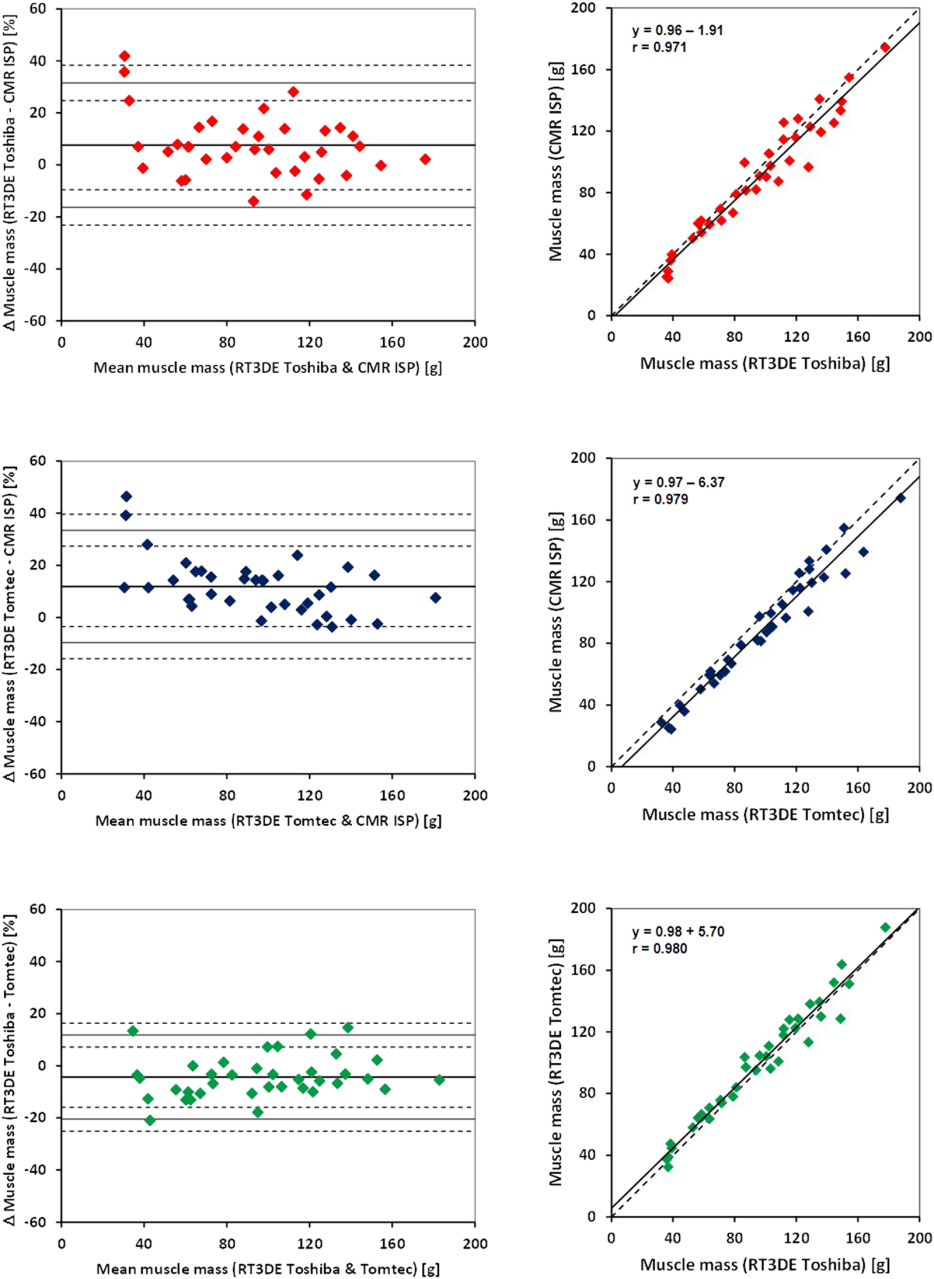
Right column: Bland and Altman plots for the comparison of left ventricular mass by type of assessment (cardiac magnetic resonance imaging vs. real-time 3-dimensional echocardiography) and software used (abscissa: mean left ventricular mass [absolute value], ordinate: differences between 2 measures [%]). Left column: Correlation between left ventricular masses (absolute values) determined by 2 types of assessment (cardiac magnetic resonance imaging *vs*. real-time 3-dimensional echocardiography) and 2 types of software. CMR ISP: Cardiac magnetic resonance, IntelliSpace Portal; CMR HDZ: Cardiac magnetic resonance HDZ MR-Tools; RT3DE, Real-time 3-dimensional echocardiography.

Bland Altman statistics of the intermodality comparison showed a minimum mean overestimation of LVM and smallest intermodality limits of agreement (LOA) as assessed by RT3DE (“Toshiba”) compared to “CMR HDZ” (Table 3, Figs 2, 3).

**Table 3:** Mean deviation [%] of the comparison of left ventricular mass of two different modalities and two software packages each.

	CMR ISP vs. CMR HDZ	RT3DE Toshiba vs. CMR HDZ	RT3DE Tomtec vs. CMR HDZ	RT3DE Toshiba vs. CMR ISP	RT3DE Tomtec vs. CMR ISP	RT3DE Toshiba vs. Tomtec
mean	−1.3	6.3	10.6	7.6	11.9	−4.3
SD	7.6	10.2	10.8	12.0	10.8	8.1
upper LOA	14.0	26.8	32.2	31.5	33.5	11.8
lower LOA	−16.5	−14.1	−10.9	−16.4	−9.7	−20.5

CMR ISP: Cardiac magnetic resonance, IntelliSpace Portal; CMR HDZ: Cardiac magnetic resonance HDZ MR-Tools; RT3DE, Real-time 3-dimensional echocardiography. LOA, limit of agreement; SD, standard deviation.

Mean intraobserver variability was <2%, and mean interobserver variability was <10% with respect to LVM as assessed by each modality and software, with closest LOA for LVM assessed by RT3DE (“Tomtec”) (Table 4).

**Table 4:** Mean inter- and intraobserver variability of left ventricular mass (LVM) [%] as assessed by each modality and software.

	CMR HDZ intra/inter	CMR ISP intra/inter	RT3DE Toshiba intra/inter	RT3DE Tomtec intra/inter
mean	1.8/9.7	−1.6/−7.9	1.8/−5.5	−0.8/5.6
SD	5.3/9.6	9.7/9.5	5.4/11.9	5.4/6.3
upper LOA	12.5/28.9	17.7/11.0	12.7/18.3	10.1/18.1
lower LOA	−8.8/−9.4	−20.9/−26.8	−9.1/−29.2	−11.7/−6.9

Inter, interobserver variability; intra, intraobserver variability; CMR ISP: Cardiac magnetic resonance, IntelliSpace Portal; CMR HDZ: Cardiac magnetic resonance HDZ MR-Tools; RT3DE, Real-time 3-dimensional echocardiography. LOA, limit of agreement; SD, standard deviation.

## Discussion

This study shows that despite software dependency and overestimation of RT3DE-assessed LVM by 5 to 10 percent, which is in the range of the interobserver variability of 2 to 10% as assessed by each modality, RT3DE favourably competes with the gold standard, CMR, in CHD patients with various forms of LV disease.

### CMR

Until now, CMR and cardiac computed tomography have been regarded as the reference standard for the calculation of LVM^5–8^. Accuracy and reproducibility of CMR-derived LVM assessment supposedly are due to homogeneous and good image contrasts that crisply define the border between myocardium and pericardium, an important reason for our finding that even despite the use of different evaluation software calculated values for CMR-derived LVM do not significantly differ. Despite observer-dependent variance, with appropriate training very high quality can be achieved in the assessment of LVM^1,9,10^.

### Echocardiography and comparison of CMR with echocardiography

M-Mode and 2D echocardiographic assessment risk inaccuracy in measurement due to oblique M-Mode sampling, foreshortening of apical views, and the fact that 2D echocardiography results are estimated based on given formulas from geometry^11–13^. Both the training background of the examiner and the exact type or version of hardware and software used dramatically influence 2D echocardiography LVM determinations^14^. Recent software allows the calculation of LVM with RT3DE^2,3,15^. On direct comparison of LVM assessment with M-Mode or 2D echocardiography and RT3DE in healthy children, RT3DE, using both manual and automated algorithms, was superior to M-Mode and 2D measurements with respect to accuracy and reproducibility. Use of an automated algorithm in particular made RT3DE superior to 2D measurement^12^. The superior accuracy of RT3DE was ascribed mainly to image-positioning errors during 2D echocardiography^12^. Even if sophisticated RT3DE analysis software cannot completely compensate for low image contrast that complicates border detection, RT3DE LVM assessment performs compellingly well even by comparison with CMR. The first studies comparing RT3DE and CMR LVM assessment, conducted in healthy adults and in adults with heart disease (mainly coronary artery disease, dilated or hypertrophic cardiomyopathy, or valve disease) as well as in healthy pediatric patients, were published in 2004^12,16–19^. Studies on patients with CHD in young adults, pediatric patients, and in infants and neonatal patients were published in 2008^2,20–22^. Most of those studies suggest that RT3DE can quantify LVM with high accuracy and reproducibility and that the technique of RT3DE and the work flow are timesaving compared with conventional CMR evaluation software^2,23^.

The main differences of our study from the cited pediatric and infant studies in CHD patients are:

1. In contrast to Laser *et al*. and Friedberg *et al*., who used *one* analyzing software each for CMR and echocardiography, in this study the influence of *different* software on LVM in 37 subjects was investigated, and CMR was compared with RT3DE^2,20^.
2. In contrast to the infant and neonate study of Friedberg *et al*., who examined intubated and anesthetized patients, all of our patients were awake^20^.
3. In contrast to the patients studied by Friedberg *et al*. (mean age 0.8 y), our patients were significantly older (mean age 18.7 y, healthy proband mean age 13.2 y), resulting in a different probe position for RT3DE (apical instead of subcostal view)^20^.
4. As in both the older studies^2,20^ no patients had global left ventricular hypertrophy or localized/asymmetric hypertrophy, but unlike Friedberg *et al*.^20^, who included a high number of patients with left-sided heart obstructive lesions and/or small LV in addition to (corrected) obstructed (±volume load) left heart lesions, we investigated a more heterogeneous cohort with structurally normal hearts as well as with LV obstruction, right-sided obstructive and/or volume-loaded lesions, or “special” cases with a single LV or with a morphologically left subpulmonary ventricle in a biventricular circulation, thus reflecting the wide clinical spectrum of heart disease^2,20^.
5. For RT3DE we used a *matrix transducer* with a lower temporal and spatial resolution than the one used by Laser *et al*.^2^.

In accordance with the results of Laser *et al*. and Friedberg *et al*., in our study direct comparison between RT3DE and CMR LVM assessment found good reproducibility^2,20^. CMR and RT3DE show satisfactory reproducibility, with LVM variability of 10% at most for RT3DE measurements, and good correlation between results obtained using different methods and assessment software tools. However, in contrast to CMR, where results of LVM were independent of the software tool used for analysis, in RT3DE the software used had a minor impact on LVM results. Thus, to date, the use of uniform software and the validation and optimization of assessment techniques are essential. We speculate that to use optimized hardware systems might yield an additional benefit, minimizing differences between RT3DE- and CMR-derived estimates of LVM^2^.

So far, the impact of assessment software has been examined with respect to ventricle volume measurement in children with and without CHD but only rarely with respect to ventricular mass (comparing manual with automated algorithms)^12,24,25^. With respect to efficacy, we explicitly opted for a semiautomated RT3DE analysis method shown to be timesaving compared with M-Mode and 2D echocardiography or conventional CMR evaluation software^2,20^.

*Trabeculated myocardium*. For the sake of homogeneity, we deviated from the previously published protocols of CMR assessment *by assigning the regions of trabeculated myocardium* to the LV cavity rather than to the LV wall as done by the echocardiographic software, thus rendering the results of the comparison of methods better comparable^5,26,27^. Even if trabeculated myocardium had not been strictly excluded from LVM calculation, for clinical purposes the results still would have been acceptably comparable: Laser *et al*. found that CMR-derived LVM increased by 5.6% if trabeculated myocardium was included to LVM calculation^2^.

### Mass

For reasons of clarity, as Friedberg *et al*. did in their study in neonates and infants with congenital heart disease^20^ and as Laser *et al*. did in a large pediatric cohort without congenital heart disease^2^, we explicitly chose to present absolute values instead of indexed values, since the former are the basis for any normalization. Since we examined patients only on one occasion we did not have to consider any changes in patients’ weight or height over time. If LVM development over time is the focus of method comparison, then normalization should be re-evaluated, especially in the populations aged <8 y and >15 y, where Sarikouch *et al*. found differences between LVM and LVM normalized to weight^26^. The reason to assess *left* ventricular muscle mass exclusively was that currently no such quantification tool is available for RT3DE with comparable accuracy in *right* ventricular muscular mass determination as has been proven for RV volumetry and ejection fraction^28^. Moreover, usually the RV wall is rather thin and the echo window is suboptimal, rendering the obtained echocardiography data difficult to process.

### Clinical implications

Once trialled and validated in a larger cohort of patients with various forms of LV disease, RT3DE LVM assessment could replace CMR examination in the *strict follow-up* of LVM with LV outflow tract obstruction or hypertrophic cardiomyopathy, and LVM overestimation could be overcome if used in combination with uniform and optimized software. For the LV that is atypical in shape, RT3DE LVM assessment must be systematically re-evaluated with respect to different hard- and software assessment tools. To this end the use of a model-based approach of RT3DE has been shown to be favorable in terms of time saving and clinical applicability^5^.

A clinical advantage of an easy-to-perform LVM assessment is improvement in prediction of events related to cardiovascular disease: According to the CARDIA study the Framingham risk score underestimates risks in young adults, while the risk score and LVM in combination are helpful independent predictors of cardiovascular disease^29^.

### Limitations

Our data are rather heterogeneous due to the small number of cases with different diagnoses, and of different age and gender; this reflects restricted access to study facilities and small numbers of patients with the same matching cardiac disease.

However, our study allows a more general assessment of methods.

At the same time, our study should be regarded as a first step towards the examination of larger cohorts with more patients with the same cardiac disease. Thus, for the establishment of RT3DE in clinical routine, further intra- and interobserver studies with larger sample sizes and the establishment of reference values for different groups of congenital heart disease are mandatory. Additionally, we quantified the whole LVM but not the septum separately, and our study lacks datasets of patients with global or localized/asymmetric cardiac hypertrophy. Thus, in clinical practice, our findings and especially the transfer of our findings to patients with disease manifest as global hypertrophy, isolated septum hypertrophy, or asymmetric hypertrophy cannot be definitely judged. Feasibility is limited as excellent image quality with excellent reflections of the pericardial border and complete data sets without dropouts are mandatory. In our study, however, also due to our investigating co-operating older patients, with an exclusion rate < 10% image quality was sufficiently good. Additionally, in hypertrophic disease with greater echogenicity, myocardial borders might be defined more easily.

## Conclusion and Outlook

Despite software dependency and overestimation of RT3DE-assessed LVM by 5 to 10 percent, RT3DE is still competitive with the gold standard, CMR, in patients with various forms of LV disease. The use of optimized software, especially for RT3DE, should improve the accuracy of LVM assessment and thereby overcome LVM overestimation.

## Methods

Within a period of 2 weeks we examined 40 subjects (19 patients with different types of CHD and 21 healthy probands; Table 1), none of them requiring general anesthesia for examination. Each subject first underwent echocardiography and within the following hour CMR.

### Real-Time 3-dimensional echocardiography (RT3DE)

**Figure 4:**
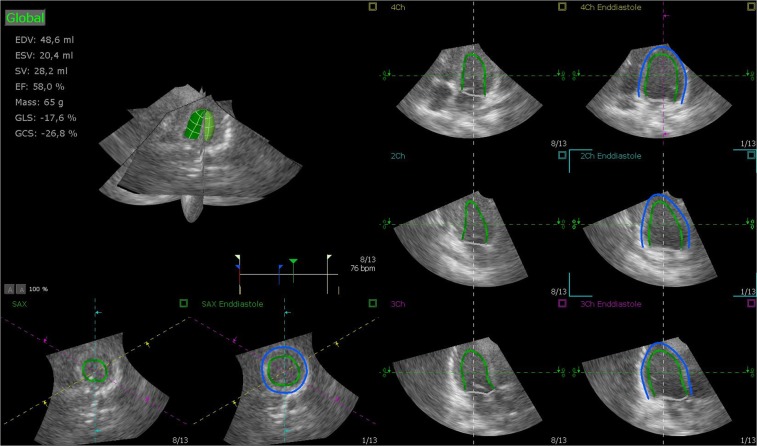
Sample images of the heart by RT3DE (software Tomtec). Two-dimensional slices from a representative real-time 3D echocardiographic data set with endocardial (green) and epicardial (blue) borders of the left ventricular myocardium.

**Figure 5:**
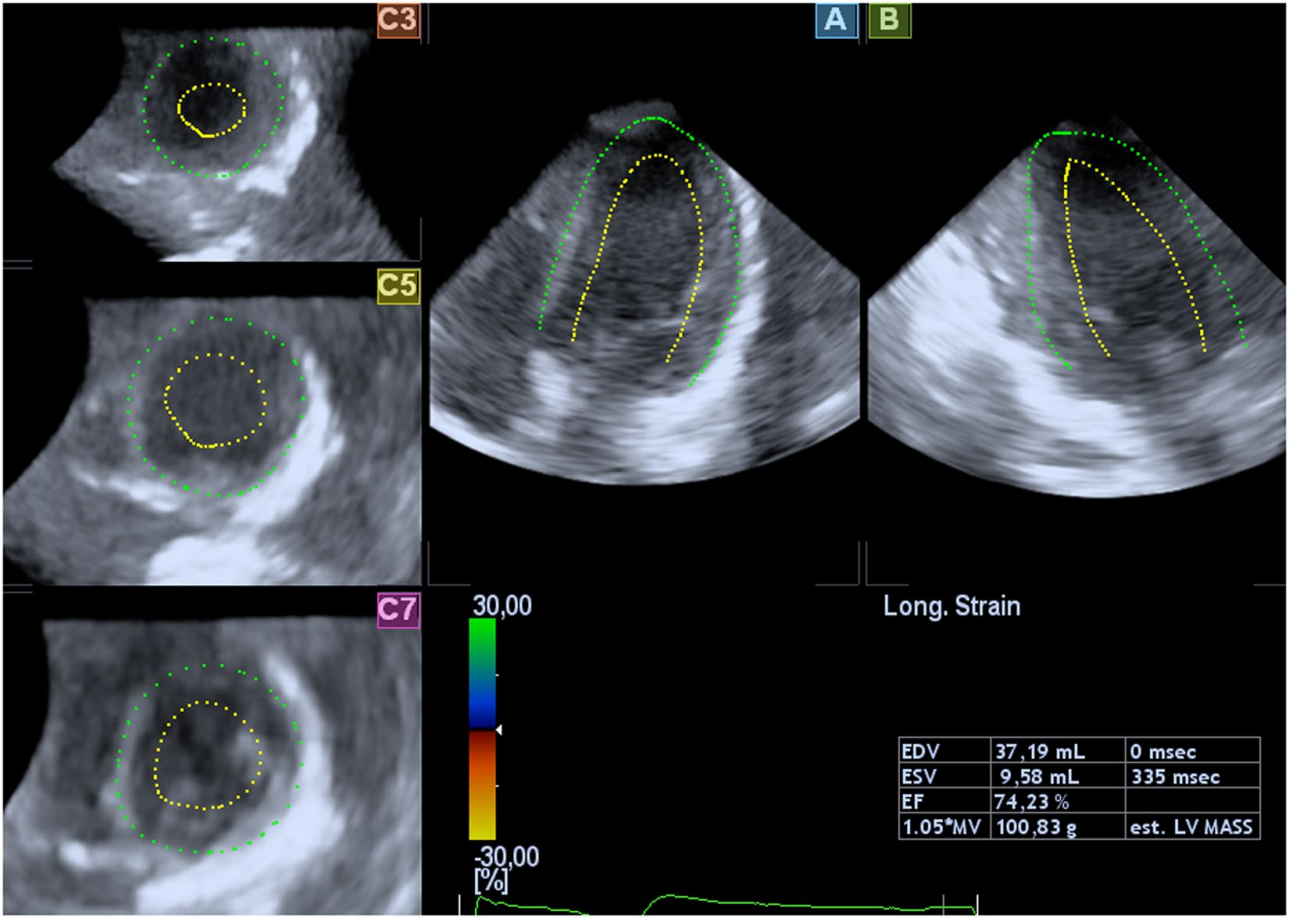
Sample images of the heart by RT3DE (software Toshiba). Two-dimensional slices from a representative real-time 3D echocardiographic data set with endocardial (yellow) and epicardial (green) borders of the left ventricular myocardium.

RT3DE was performed by one experienced sonographer (KTL) using Artida (Artida 4D-System, Toshiba Medical Systems, Neuss, Germany, transducer PST25-TX). The examination consisted of a standard 3D protocol, including left-sided position of the patient, apical view with the apex next to the transducer including the entire LV portion in the 2D planning view using 4 subvolumes at 90° to 110°, and 4-beat acquisition during one end-expiratory breath-hold^2^. All RT3DE data of LVM were analyzed using two different software packages: i) “Toshiba” (Wall Motion tracking system, Toshiba Medical Systems, Neuss, Germany) and ii) “Tomtec” (Image Arena platform [Version 4.6, built 4.6.2.12; TomTec, Unterschleissheim, Germany], 4D LV-Analysis Version 3.1 [built 3.1.0.258661]). After a first automated contour-tracking process, endocardial and epicardial borders were manually re-drawn. Myocardial mass was calculated as myocardial volume between the endocardial and epicardial border multiplied by myocardial density (1.053 g/ml) (Figs 4, 5)^2,20^. LVM was not calculated if the borders were not adequately seen. The summation of disc method was used in both evaluation tools for CMR data (see below), allowing direct comparison of methods^20^.

### Cardiac magnetic resonance imaging (CMR)

Assessment of LVM was done using a multi-transmit 3.0 Tesla magnetic resonance imaging system (Achieva 3.0 T TX, Philips Healthcare, Best, The Netherlands). We used a published standard protocol^5,26,27^: Maximum gradient performance was 80 mT/m, slew rate 200 T/m/s. For signal detection a 32-element phased-array receive-only surface coil was used. Typically, 14–23 axial slices with no slice gap were collected applying a multi-slice multi-phase vector electrocardiogram-triggered steady-state free precession gradient-echo sequence. Pulse repetition time/echo time/excitation angle were 2.7 ms/1.35 ms/40°, slice thickness of 5–6 mm, matrix size of 160 × 240 encompassing a field-of-view of 384 mm. This setup results in a spatial in-plane resolution of 1.6 × 1.6 mm². By default, 25 cardiac phases were acquired under breath-holding periods of below 12 seconds duration.

**Figure 6:**
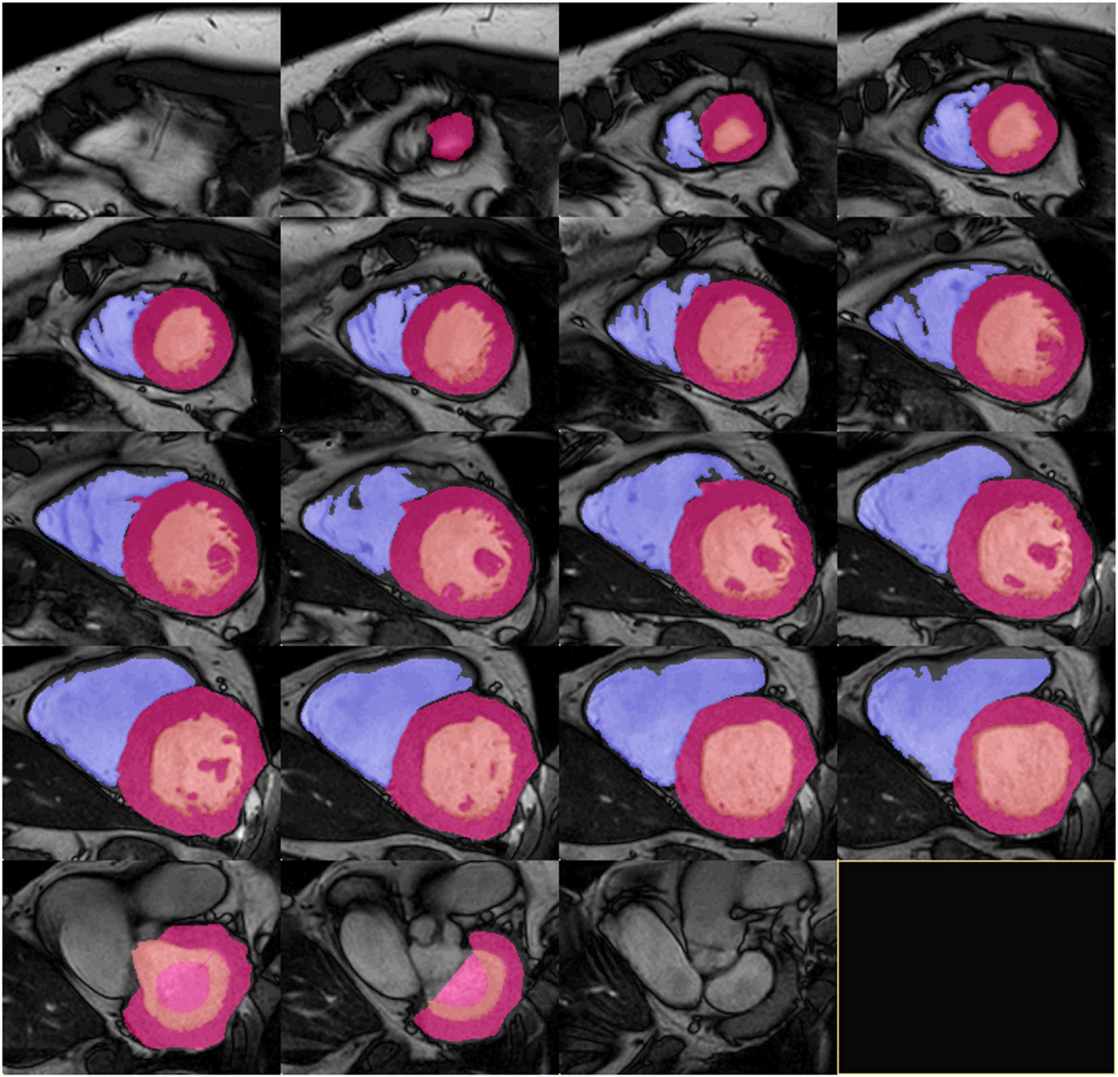
Sample images of the heart by CMR (software HDZ). Two-dimensional slices from a representative CMR data set with left ventricular mass tagged in pink, left ventricular lumen tagged in rose, and right ventricular lumen tagged in violet.

**Figure 7:**
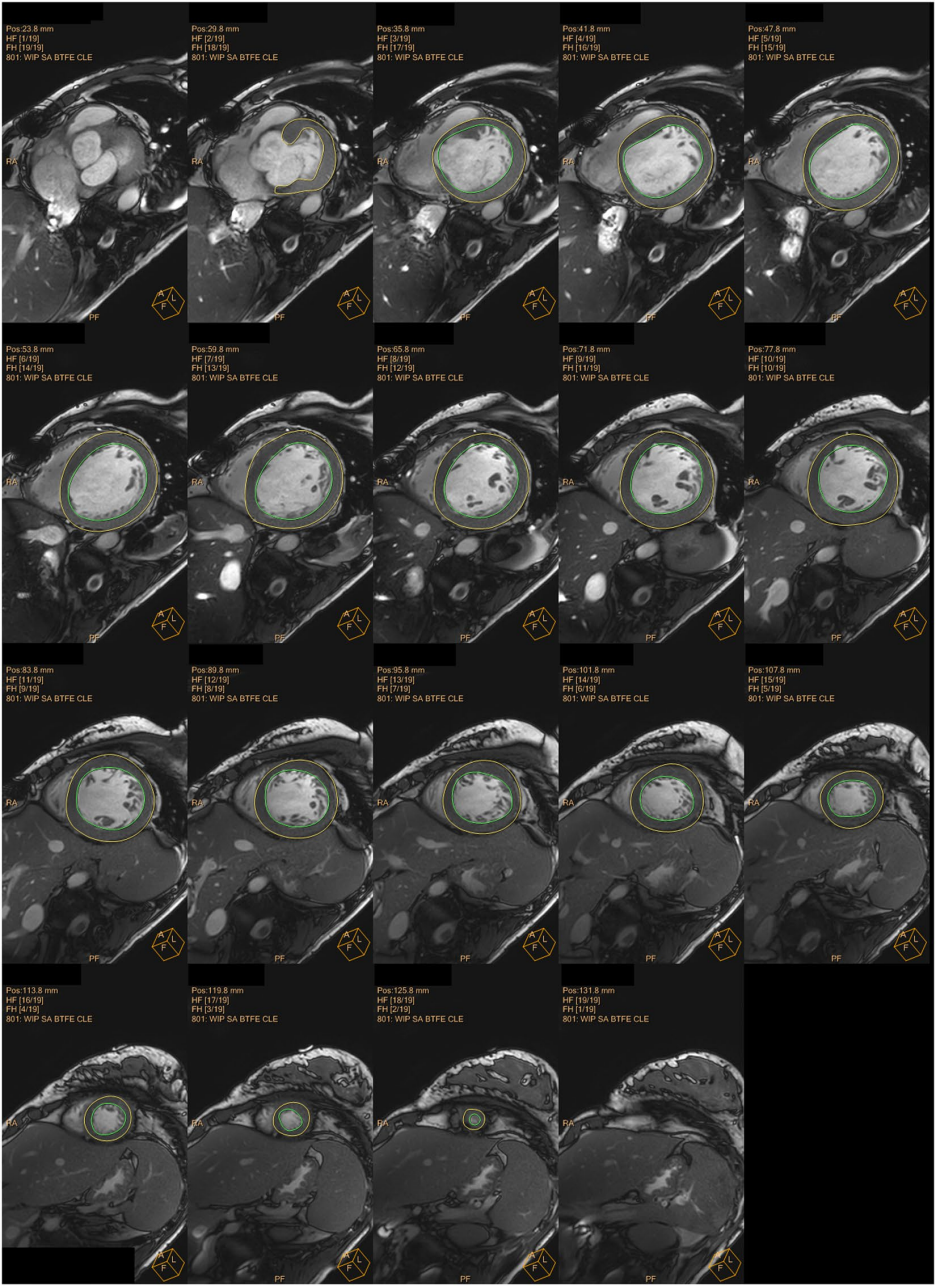
Sample images of the heart by CMR (software ISP). Two-dimensional slices from a representative CMR data set with endocardial (green) and epicardial (yellow) borders of the left ventricular myocardium.

Image data were transferred to external computer workstations for assessment of LVM. End-diastolic images were used to define LVM in each case by applying 2 different software packages: i) a dedicated customized CMR-Tool labelled “CMR HDZ” (HDZ MR-Tools; R2015, HDZ, Bad Oeynhausen, Germany); ii) CMR Analysis tool labelled “CMR ISP” (IntelliSpace Portal, Release v7.0.1; Philips Healthcare, Best, The Netherlands). Both software systems used threshold-based semiautomated detection of endocardial/vascular borders. Papillary muscles were assigned as an exemption to the blood pool to be comparable with the echocardiographic analysis (Figs 6, 7).

### Reproducibility

Data were evaluated by two experienced observers. Analysis of RT3DE was performed blinded to CMR data, and *vice versa*. To define reproducibility, intra- and interobserver variability was determined in 10 randomly selected data pairs with results blinded. For intraobserver reliability testing, >2 weeks after the first analysis a blinded second analysis was done by the same observer.

### Informed consent

Informed consent for study participation was obtained from each participant or from his parent and/or legal guardian in case of participants under the age of 18 y.

### Statistical analysis

Statistical analysis was done using the Statistical Software Package SPSS 20.0 (SPSS, Chicago, IL). The Shapiro-Wilk test was used to test LVM data on normal distribution, and Levene statistics were used to evaluate variance homogeneity. Comparison of LVM as assessed by the 2 different software analysis systems for echocardiography and CMR each and between the different modalities was performed using paired Student’s t-test for normally distributed data; otherwise the Wilcoxon test was used. Agreement between CMR and echocardiographic LVM and the different software packages, as well as intra- and interobserver variability, was calculated using the ICC and by Bland-Altman-analysis^30^. A p-value of < 0.05 was considered sufficient for clinical significance.

### Graphics

Graphics were created using Microsoft Office Professional Plus 2010 (Excel and Power Point) and using Metapost, a free interpreter software by John D. Hobby, version 1.211, distributed within the TEX framework via the public domain Comprehensive TEX Archive Network.

### Ethical approval and informed consent

The study protocol was approved by the local ethics committee (ethics committee of the Medical faculty of the Ruhr University Bochum, section Bad Oeynhausen, registration number AZ 9/2011), the methods were carried out in accordance with the relevant guidelines and regulations, and the subjects gave written informed consent.

## Data Availability

All data generated or analyzed during this study are included in this article.
